# Pre-metazoan origins and evolution of the cadherin adhesome

**DOI:** 10.1242/bio.20149761

**Published:** 2014-11-13

**Authors:** Paul S. Murray, Ronen Zaidel-Bar

**Affiliations:** 1 Department of Biochemistry and Molecular Biophysics, Columbia University, New York, NY 10032, USA; 2 Center of Computational Biology and Bioinformatics, Department of Systems Biology, Columbia University, Irving Cancer Research Center, New York, NY 10032, USA; 3 Mechanobiology Institute Singapore, National University of Singapore, Singapore 117411; 4 Department of Biomedical Engineering, National University of Singapore, Singapore 117575

**Keywords:** cadherin, adherens junction, multicellularity, evolution, protein interaction network

## Abstract

Vertebrate adherens junctions mediate cell–cell adhesion via a “classical” cadherin–catenin “core” complex, which is associated with and regulated by a functional network of proteins, collectively named the cadherin adhesome (“cadhesome”). The most basal metazoans have been shown to conserve the cadherin–catenin “core”, but little is known about the evolution of the cadhesome. Using a bioinformatics approach based on both sequence and structural analysis, we have traced the evolution of this larger network in 26 organisms, from the uni-cellular ancestors of metazoans, through basal metazoans, to vertebrates. Surprisingly, we show that approximately 70% of the cadhesome, including proteins with similarity to the catenins, predate metazoans. We found that the transition to multicellularity was accompanied by the appearance of a small number of adaptor proteins, and we show how these proteins may have helped to integrate pre-metazoan sub-networks via PDZ domain–peptide interactions. Finally, we found the increase in network complexity in higher metazoans to have been driven primarily by expansion of paralogs. In summary, our analysis helps to explain how the complex protein network associated with cadherin at adherens junctions first came together in the first metazoan and how it evolved into the even more complex mammalian cadhesome.

## INTRODUCTION

Adherens junctions (AJs) are multi-protein structures that form a bridge between the actin cytoskeletons of adjacent cells, enabling their integration into higher order organizations such as epithelial sheets. In this capacity, they play a critical role in coordinating cellular mechanics and tissue dynamics during development and homeostasis, as well as in diseases such as cancer (reviewed by Harris and Tepass, 2010). Adhesion between cells by AJs in vertebrates is primarily mediated by transmembrane receptors of the type I “classical” cadherin family. The structure and function of vertebrate “classical” cadherin extracellular regions is now well understood (reviewed by Brasch et al., 2012). Briefly, the cadherin ectodomain is comprised of five consecutively-linked extracellular-cadherin (EC) domains. Binding between apposed cells is primarily, but not exclusively, homophilic and is dependent on Ca^2+^. Trans- and cis-interactions of the ectodomains facilitate the oligomerization of cadherins into higher order structures, which are further stabilized by interactions of the cytoplasmic domain, and its binding partners, with F-actin (Harrison et al., 2011; Hong et al., 2013).

The cytoplasmic domain, or tail, of “classical” cadherins is approximately 150 amino acids long and natively unstructured (Huber et al., 2001). Within the tail, tandem unstructured domains, the juxtamembrane domain (JMD) and catenin-binding domain (CBD), bind P120 and β-catenin, respectively (Thoreson et al., 2000; Stappert and Kemler, 1994). Both P120 and β-catenin belong to the armadillo (ARM) repeat family of proteins, which is characterized by forty-amino acid repeats of three α-helices (Huber and Weis, 2001; Ishiyama et al., 2010). An electro-positive groove formed by tandem ARM repeats serves as a binding site for the JMD and CBD, which can be postranslationally modified at multiple sites; depending on the site, the interaction between cadherin and catenins is either strengthened or weakened (Dupre-Crochet et al., 2007). α-catenin interacts with β-catenin (Pokutta and Weis, 2000), and may link the complex to the actin cytoskeleton (Desai et al., 2013; Drees et al., 2005). The binding of cadherins to catenins appears to be a feature that is common to all metazoans, from vertebrates to sponge, but absent from pre-metazoans (Nichols et al., 2012; Miller et al., 2013). Herein, we refer to any cadherin that contains both EC domains and a CBD as “classical”-like.

Like metazoans, the choanoflagellates, which are the closest extant unicellular relatives of metazoans, contain many cadherins (Nichols et al., 2012; King et al., 2008; Abedin and King, 2008; reviewed by Richter and King, 2013). However, the two choanoflagellates with sequenced genomes, *Salpingoeca rosetta* and *Monosiga brevicollis*, do not contain a “classical”-like cadherin in which a clearly defined JMD or CBD can be recognized in the cytoplasmic tail. Choanoflagellate cadherins are not known to participate in cell–cell adhesion and little is known about their function in these organisms (Dayel et al., 2011; Fairclough et al., 2013), though two cadherins from *M. brevicollis* have been localized to the feeding collar (Abedin and King, 2008). The slime mold, *Dictyostelium discoideum*, has proteins related to β- and α-catenin (Aardvark and *Dd*α-catenin, respectively), which form a complex and are important in establishing epithelial-like cell polarity, along with *Dictyostelium* IQGAP (rgaA) and myosin II (Dickinson et al., 2012b; Dickinson et al., 2011; Grimson et al., 2000). Though Aardvark and *Dd*α-catenin are not known to participate in cell–cell adhesion, they share many functional similarities with β-catenin and α-catenin, respectively, and are therefore considered close relatives of these catenins (reviewed by Miller et al., 2013). Aardvark-like proteins have also been reported in the choanoflagellate *S. rosetta* and in the filasterea *Capsaspora owczarzaki* (Nichols et al., 2012; Suga et al., 2013). Thus, at least some unicellular holozoa contain both cadherins and proteins related to the catenins (Aardvark), but there is currently no evidence for a physical interaction between the two protein families in these unicellular organisms.

P120, β- and α-catenin serve as hubs for a diverse network of associated proteins (reviewed by Braga, 2002; Hartsock and Nelson, 2008; Stepniak et al., 2009; Cavallaro and Dejana, 2011; Bertocchi et al., 2012; Ratheesh and Yap, 2012). In addition to providing physical connections with the cytoskeleton, the network of proteins associated with AJs regulates the location and strength of adhesion complexes in response to internal and external signals and forces (reviewed by Lecuit, 2005; Baum and Georgiou, 2011; Niessen et al., 2011). We refer henceforth to this larger functional network, including the cadherin–catenin “core”, as the “cadherin adhesome” or “cadhesome”. Based on defined criteria and a systematic analysis of the literature, we previously compiled a list of all known human cadhesome components and their interactions (Zaidel-Bar, 2013). The literature-based cadhesome has 173 proteins, which can interact with each other in up to ∼400 different combinations, and is populated by a variety of structural, catalytic, and regulatory proteins. Our goal here has been to understand which cadhesome components were incorporated from pre-metazoan systems, which were innovated at the onset of multicellularity, and which types of components expanded in higher metazoans. Starting with the cadherin–catenin “core” of AJs, we then expand our analysis to the larger cadhesome network, tracing its evolution from pre-metazoans to vertebrates, both in terms of its components and functional interactions.

## MATERIALS AND METHODS

### Reciprocal best-hits analysis

**Fig. 1.**
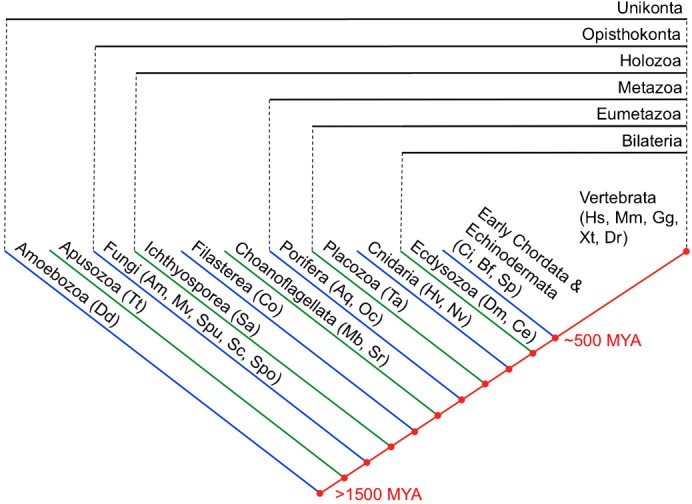
Phylogeny of metazoans and their uni-cellular ancestors. The phylogenetic relationship between the twenty-six metazoan and pre-metazoan species we analyzed is depicted. Each species is positioned according to its phylum or kingdom; species are also grouped into broader taxonomic categories, delimited by black bars. “MYA” stands for million years ago.

To identify analogous proteins with similar protein domain architecture to the 173 human cadhesome components, we performed a reciprocal best-hits analysis of twenty-five unikont (the unity of holozoa, fungi, and amoebozoa/apusuzoa) genomes: *Homo Sapiens* (Hs); *Mus musculus* (Mm); *Gallus gallus* (Gg); *Xenopus tropicalis* (Xt), *Danio rerio* (Dr); *Ciona intestinalis* (Ci); *Branchiostoma floridae* (Bf); *Strongylocentrotus purpuratus* (Sp); *Drosophila melanogaster* (Dm); *Caenorhabditis elegans* (Ce); *Hydra vulgaris* (Hv); *Nematostella vectensis* (Nv); *Trichoplax adhaerens* (Ta); *Amphimedon queenslandica* (Aq); *Oscarella carmela* (Oc); *Monosiga brevicollis* (Mb); *Salpingoeca rosetta* (Sr); *Sphaeroforma arctica* (Sa); *Capsaspora owczarzaki* (Co); *Allomyces macrogynus* (Am); *Mortierella verticillata* (Mv); *Spizellomyces punctatus* (Spu); *Saccharomyces cerevisiae* (Sc); *Schizosaccharomyces pombe* (Spo); *Thecamonas trahens* (Tt); and *Dictyostelium discoideum* (Dd). These species were selected for analysis because they span the ancient to recent ancestry of metazoans, including critical innovations such as cell–ECM and cell–cell adhesion, and their complete or draft genomes are available (Ruiz-Trillo et al., 2007). The phylogenetic relationship between the twenty-five species and *Homo sapiens* is depicted in Fig. 1.

The reciprocal best-hits analysis was carried out as follows: 1) each of the 173 human cadhesome components was used as a protein BLAST (Altschul et al., 1997) query in a search of the sequence databases of twenty-five organisms (Ruiz-Trillo et al., 2007; Apweiler et al., 2004; Hemmrich and Bosch, 2008). 2) The sequence with the lowest E-value was retrieved for each query, from every organism, if the E-value was below 10 (bit score of ∼30). 3) Each qualifying hit was submitted to both SMART (Letunic et al., 2012; Schultz et al., 1998) and CDD (Marchler-Bauer et al., 2013; Marchler-Bauer et al., 2011) in order to determine its protein domain content. The two databases are largely redundant, but in a number of cases proved to be complementary. 4) Each identified sequence was used as a BLAST query in a reciprocal search (re-BLAST) of the human sequence database. A protein was considered as analogous if it retrieved the original human component, or a highly related component, in the re-BLAST step, and was comprised of the same major domains – hence the terminology “reciprocal best-hit”.

We also applied the reciprocal best-hits approach to the sponge and *T. adhaerens* analogous proteins we identified when we searched with the human cadhesome, using them to BLAST search all pre-metazoans, then re-BLAST search the pre-metazoan sequences to humans. Similarly, we used all unicellular holozoa analogous proteins identified in BLAST searches with human, sponge, or *T. adhaerens* analogous proteins to BLAST search all pre-metazoans, then re-BLAST against humans. These added levels of searching identified many analogous proteins that were not identified with the human analogous proteins alone, e.g. *Dictyostelium* sibC, which we identified in a search with *O. carmela* integrin, and has been shown experimentally to function in a manner similar to vertebrate integrin (Cornillon et al., 2006). In general, analogous proteins had a high bit score/low E-value and high percent query coverage in the BLAST round, but this was not always the case (e.g. *Dd*Aardvark, which has been experimentally defined as related to β-catenin (Dickinson et al., 2011)). Since AJs might differ significantly across species, our primary objective was to determine whether or not the fundamental building blocks of the human junction exist. A full list of analogous proteins in each of the organisms can be found in supplementary material Table S4.

“Classical” and “classical”-like cadherins differ significantly in their extracellular regions from organism to organism (Oda and Takeichi, 2011; Hulpiau and van Roy, 2009; Hulpiau and van Roy, 2011). However, all share a conserved cytoplasmic tail that binds catenins. For this reason, we queried the organism sequence databases with both A) full-length type I and II human cadherins, and B) their cytoplasmic tails alone. This search method resulted in good coverage of both “classical” and “classical”-like cadherins in all organisms analyzed. We then also searched all pre-metazoans with the full-length “classical”-like cadherins from all the non-vertebrate metazoans we analyzed, as well as their cytoplasmic tails alone.

Searching choanoflagellata, filasterea and ichthyosporea with the P120 and β-catenin families identified sequences that were not readily identified by the re-BLAST step as from a known human family. Many of the same sequences were retrieved when we BLAST searched choanoflagellata, filasterea and ichthyosporea with *Dictyostelium* Aardvark. In order to better characterize these sequences, we used each in a re-BLAST search of slime mold, yeast, and the green algae, *Volvox carteri*. We also used each to search for similar sequences in the other unicellular holozoa. We iteratively performed this BLAST, re-BLAST process until each sequence could be confidently defined by its sequence similarity and domains as related to the catenins/Aardvark, members of a known family (e.g. importin-α), or unrelated to the catenins/Aardvark. This all-against-all BLAST search of eight species helped to characterize many of the choanoflagellata, filasterea and ichthyosporea proteins as Aardvark-like; it also identified in them many sequences in addition to the original hits based on our BLAST search with the human proteins (supplementary material Table S5).

### Sequence analysis and structure prediction

SMART and TMHMM (Krogh et al., 2001; Sonnhammer et al., 1998) were used to predict transmembrane helices, other domains in the cytoplasmic tail, and membrane topology of the metazoan “classical”-like cadherins, the 23 *M. brevicollis* and 29 *S. rosetta* cadherins, and the two *C. owczarzaki* and *T. trahens* cadherins (supplementary material Table S4). Cadherin PDZ-binding motifs were identified via visual inspection. The T-coffee suite of methods (Di Tommaso et al., 2011; Notredame et al., 2000) was used to produce multiple sequence alignments.

Using BLAST, SMART, and CDD, it was not always clear how many ARM repeats were formed by the choanoflagellata, filasterea and ichthyosporea Aardvark-like sequences. Therefore, we used a number of additional sequence- and structure-based methods to predict ARM repeats, including Superfamily (Gough et al., 2001), ARD (Palidwor et al., 2009), HHrepID (Biegert and Söding, 2008), and secondary structure prediction (McGuffin et al., 2000; Pollastri et al., 2002; Cole et al., 2008). We also used the servers Pudge (Norel et al., 2010), PHYRE2 (Kelley and Sternberg, 2009), and I-tasser (Zhang, 2008; Roy et al., 2010) to predict the tertiary structure of the sequences. Only in the union of these methods were we able to confidently predict ARM repeats (supplementary material Table S6). For all modeled sequences, there was good agreement between Pudge, PHYRE2, and I-tasser, in terms of template selection, and query to template alignment. We scored all the models constructed with a number of model evaluation tools: Prosa (Sippl, 1993), DFIRE (Zhou and Zhou, 2002), and Verify3D (Bowie et al., 1991; Lüthy et al., 1992) (supplementary material Table S7).

### Domain architecture-based clustering

The 173 human cadhesome components were first clustered into families, by eye, based on matching SMART- and CDD-predicted domains. For example, we clustered the membrane-associated guanylate kinase (MAGUK) proteins, ZO-1 (TJP1) and ZO-2 (TJP2) in the same group because they contain the same number and order of domains while other MAGUK proteins, e.g. MAGI1 were assigned to separate families. This clustering yielded a “simplified” cadhesome, consisting of 112 protein families. A full list of the protein clusters in the “simplified” cadhesome can be found in supplementary material Table S4. Using the “simplified” cadhesome, we determined when, in the evolutionary timeline suggested by the species analyzed here, families of proteins appeared. To analyze paralog expansion, we clustered components the same as the “simplified” cadhesome, except for ACTN1/4/SPTBN1 and the MAGUK proteins, which were more broadly clustered based on similar, but not exact type, number, and order of domains.

## RESULTS

### Conserved motifs in the cytoplasmic tail of metazoans cadherins

**Fig. 2.**
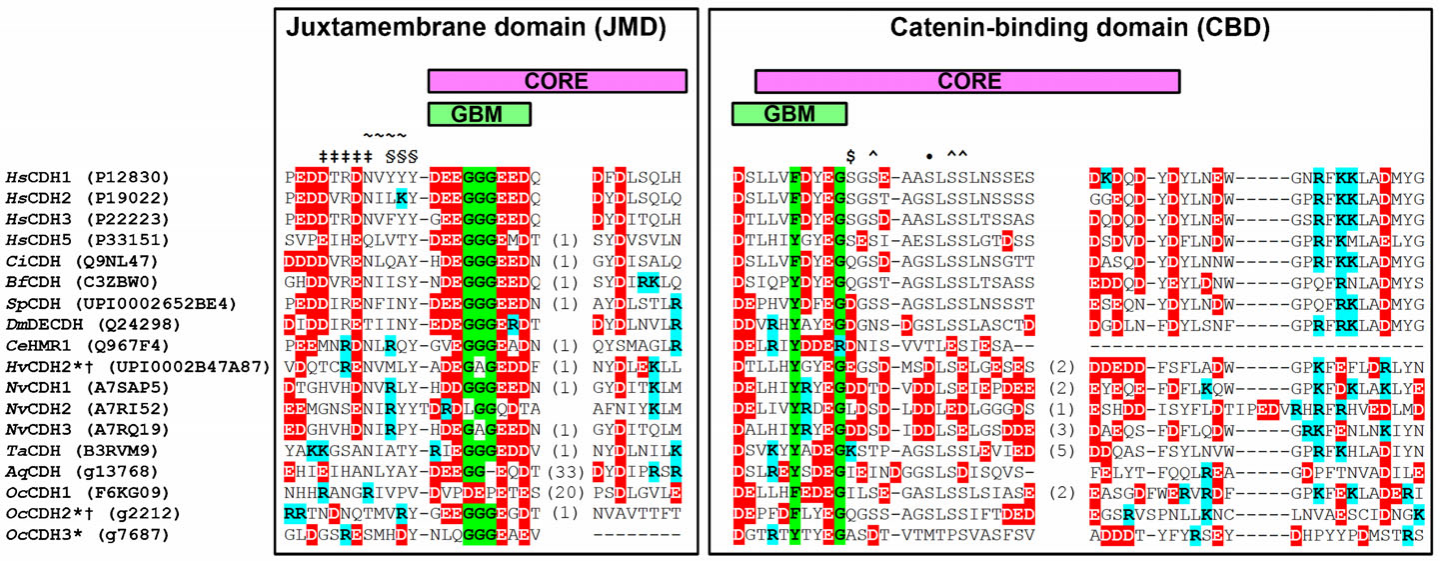
Sequence alignment of cadherin cytoplasmic domains from metazoa. The cytoplasmic tails of the metazoan “classical”-like cadherins are aligned with human E-cad (*Hs*CDH1), and divided up into JMD and CBD. Magenta bars indicate core regions known via experiment to be required for cadherin–catenin binding. Green bars indicate groove-binding motifs (GBMs), which are known to bind in an extended peptide conformation to the ARM repeats of catenins, based on structures of E-cad-P120 and E-cad-β-catenin. If inserts exist relative to E-cad, the number of intervening amino acids is indicated. Cadherins denoted by “*” are named out of convenience, as no general naming standard exists (UniProt or Compagen identifiers are also included). “†” indicates that the protein has no EC domains. Residues are colored according to their properties, with acidic residues (E/D) in red and basic residues (K/R) in cyan. Amino acids in the JMD and CBD are annotated based on known functions in vertebrates: “‡” indicates a CASP3 cleavage site; “∼” a NUMB PTB-binding site; “§” a Hakai binding site; “•” a CSNK1 phosphorylation site; “$” a CSNK2 site, and “**∧**” a GSK3β site.

Since a large part of the cadhesome involves binding or regulating the cytoplasmic tail of cadherin we started our evolutionary investigation by examining the degree of conservation of various motifs along the tail of cadherin. To this end we performed BLAST searches with human “classical” cadherins against the genomes of twenty-five species ranging across unikonts (Fig. 1). Our searches retrieved “classical” or “classical”-like cadherins in all metazoans analyzed. Fig. 2 shows the alignment of the cytoplasmic tails of some of these cadherins, from human E-cad (*Hs*CDH1) at the top, down to the most basal metazoans, the sponges *Oscarella carmela* and *Amphimedon queenslandica*, in order of increasing evolutionary age. The full-length JMD and CBD span most of the length of the “classical” cadherin tail, but we show only the most crucial catenin-binding regions, as indicated in the figure. Depicted as magenta bars are two regions, the JMD core and CBD core, which are known via mutagenesis to be absolutely required for binding P120 and β-catenin, respectively (Thoreson et al., 2000; Stappert and Kemler, 1994). Crystal structures of E-cad JMD-P120 (Ishiyama et al., 2010) and E-cad CBD-β-catenin (Huber and Weis, 2001) demonstrate that, within both core regions, a motif of approximately ten residues (green bars) binds in the electro-positive groove formed by the ARM repeats of its cognate catenin. Mutagenesis of residues in P120 and β-catenin that contact these motifs abrogates cadherin binding, which suggests that they are crucial to this interaction (Ishiyama et al., 2010; Graham et al., 2000). We refer to these two motifs, xx[ED]GGGExx (where “x” is any amino acid) in the JMD, and 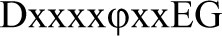 (where “
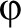
” is aromatic) in the CBD, as groove binding motifs (GBMs).

Three consecutive glycines in the JMD GBM anchor the region in a small hydrophobic pocket in the ARM repeats of P120, while acidic residues on both sides contact basic residues in P120 (Ishiyama et al., 2010). In the GBM of the CBD, a phenylalanine anchors the region in a small pocket of β-catenin, while acidic residues on both ends contact basic residues in β-catenin (Huber and Weis, 2001). Thus, both GBMs use a combination of acidic residues and glycine/hydrophobic residues to bind catenins. Fig. 2 demonstrates that the acidic catenin-binding residues of both the JMD GBM and CBD GBM are well conserved in metazoa, as are the intervening glycine/hydrophobic residues. As noted previously, the known “classical”-like cadherin in *O. carmela* (*Oc*CDH1) has a functioning CBD, but no apparent JMD (Nichols et al., 2012). However, we found at least one sequence in *O. carmela* (*Oc*CDH2*) that contains both a JMD GBM and CBD GBM. Though *Oc*CDH2* lacks EC domains, it does contain EGF and LamG domains, which suggests it is related to early metazoan cadherins. Thus, both GBMs appear together in at least one sequence from each sponge, which suggests that both innovations were present in tandem in the last common ancestor of the sponges and other metazoans.

Most of the residues in the E-cad JMD and CBD do not form secondary structure (Ishiyama et al., 2010; Huber and Weis, 2001). The “classical”-like cadherins we analyzed also have a low propensity to form secondary structure, and are devoid of SMART-predicted globular domains (supplementary material Table S4). Such long, unstructured regions leave the E-cad JMD and CBD available for posttranslational modification. For example, binding of β-catenin is inhibited by CSNK1 phosphorylation of human E-cad Ser844 (designated by “•” in the CBD of Fig. 2A) (Dupre-Crochet et al., 2007), which we found to be well conserved in metazoans. CSNK2 phosphorylates Ser838 (“$”) (Serres et al., 2000), which enhances binding to β-catenin, but this serine is not conserved outside of vertebrates. We found at least one GSK3β phosphorylation site (“**∧**”) (Huber and Weis, 2001) to be conserved in all metazoans we analyzed. The presence of known sequence elements in the cadherins from a wide variety of metazoans suggests that a subset of phospho-regulatory switches were present in the AJs of the most ancestral metazoans. This contrasts with other regulatory sites in the JMD, which we found to be later innovations: a CASP3 apoptotic cleavage site ([DSTE]xxD[GSAN], “‡”) (Steinhusen et al., 2001); a NUMB PTB domain-binding site (NVYY, “∼”) (Wang et al., 2009); and a YYY motif (“§”), which binds the ubiquitin ligase Hakai (CBLL1) (Mukherjee et al., 2012; Fujita et al., 2002).

Previous studies based on searches for EC domains have identified numerous cadherins in choanoflagellata and filasterea: 23 in *M. brevicollis*, 29 in *S. rosetta*, and one in *C. owczarzaki* (Abedin and King, 2008; Nichols et al., 2012). However, using SMART, we found no “classical” cadherin tail in any of these cadherins (supplementary material Table S4). Furthermore, we found no JMD core, CBD core, JMD GBM (xx[ED]GGGExx), or CBD GBM (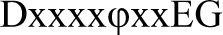) when we visually inspected the known unicellular holozoa cadherins, as well as a second cadherin in *C. owczarzaki* (E9CEE8) and two cadherins in *Thecamonas trahens* (AMSG_00600 and AMSG_08522) that we identified here for the first time. Interestingly, we found PDZ-binding motifs in some of the choanoflagellate cadherins, similar to metazoan cadherins (supplementary material Table S4) (Demontis et al., 2006).

### β-catenin/Aardvark and the *Dictyostelium* polarity network in unicellular holozoa

**Fig. 3.**
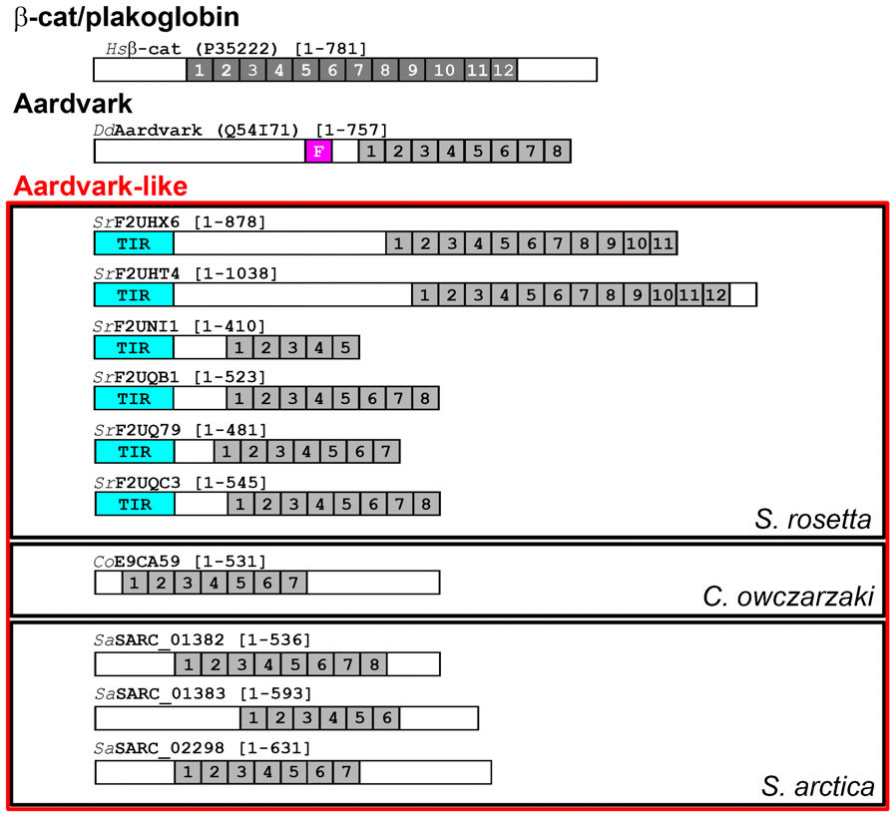
Aardvark-like analogous proteins in choanoflagellata, filasterea, and ichthyosporea. Unicellular holozoa sequences identified as related to Aardvark/β-catenin are depicted in relation to vertebrate β-catenin and Aardvark. ARM repeats are numbered and represented as boxes in the context of the full sequence, with structurally resolved repeats colored dark grey and predicted repeats colored light grey (see Materials and Methods). Boxes are also used to represent other predicted domains, which are labeled and colored: “F” is F-box and “TIR” is toll interleukin-1 receptor. Species of origin, identifier (Uniprot/Broad Institute gene), and length of each sequence are also depicted.

In order to determine if pre-metazoans have ARM repeat proteins related to β-catenin/Aardvark, we performed BLAST searches using human β-catenin and plakoglobin (JUP) and *Dictyostelium* Aardvark as queries. Our searches retrieved a variety of ARM proteins with significant sequence similarity (high BLAST E-value) to *Dd*Aardvark in *C. owczarzaki* (*Co*E9CA59) and *S. rosetta*, consistent with previous work (Nichols et al., 2012), and also in *Sphaeroforma arctica* (*Sa*SARC_01382, *Sa*SARC_01383 and *Sa*SARC_02298) (Fig. 3). In total we found at least six sequences similar to Aardvark in *S. rosetta* (*Sr*F2UHX6, *Sr*F2UHT4, *Sr*F2UNI1, *Sr*F2UQB1, *Sr*F2UQ79 and *Sr*F2UQC3), only one of which was previously identified. We found no proteins analogous to Aardvark in the other choanoflagellate we analyzed, *M. brevicollis*. Consistent with *Dd*Aardvark, all but one of these sequences (*Sa*SARC_01383) returns ARMC6 in the re-BLAST search of humans, and Aardvark in a re-BLAST search of *Dictyostelium*, the latter with a higher E-value (supplementary material Table S5). To further characterize these sequences, we employed a number of additional sequence- and structured-based methods (see Materials and Methods). Though the choanoflagellata, filasterea and ichthyosporea sequences lack an F-box domain, and some have added Toll/interleukin-1 receptor (TIR) domains, our analysis suggests their ARM repeats have significant sequence similarity to *Dd*Aardvark. Hence, we refer to these ten unicellular holozoa sequences as Aardvark-like.

Together with *Dd*α-catenin, IQGAP1 (rgaA), and myosin II, *Dictyostelium* Aardvark helps to establish cell polarity in this organism (Dickinson et al., 2012b; Dickinson et al., 2011). In addition to Aardvark-like sequences, each of the unicellular holozoa we analyzed also possessed proteins analogous to α-catenin or the closely-related vinculin, IQGAP, and myosin II. Thus, *S. rosetta*, *C. owczarzaki*, and *S. arctica*, all seem to have conserved the components of the *Dictyostelium* polarity network. Furthermore, using *S. rosetta* transcriptomics data (Fairclough et al., 2013) we found that one of the Aardvark-like proteins we identified (F2UQ79), α-catenin/vinculin (F2UM91), IQGAP (F2TWA6), and myosin II (F2U9L1) are all upregulated in thecate cells (supplementary material Table S1), which suggests that these four proteins function together in this organism, perhaps to establish polarity in a manner akin to *Dictyostelium*. As in *D. discoideum*, the binding partner of the *S. rosetta* Aardvark ARM repeats is not known, but it is interesting to note that six cadherins were also found to be upregulated in thecate cells (Fairclough et al., 2013).

Compared to rgaA, the proteins analogous to IQGAP from *S. rosetta*, *C. owczarzaki*, and *S. arctica* have gained the WW and IQ domains (supplementary material Table S4), which suggests that more of its ten known vertebrate interactions may have been in place prior to multicellularity. In this way, incorporation by metazoans of a single adaptor, e.g. Aardvark/β-catenin, and its pre-metazoan interaction network may have added the functionality of multiple proteins to the newly-formed AJ. Proteins analogs of the *Dictyostelium* polarity network are also present in all the metazoans we analyzed (except *Drosophila* that is missing an IQGAP1; other insects, such as honey bees, have IQGAP1 analogs). In the context of vertebrate AJs, IQGAP binds β-catenin and myosin (Weissbach et al., 1998; Fukata et al., 1999) and can be either a positive or negative regulator of E-cadherin-mediated adhesion (reviewed by Noritake et al., 2005).

### Incorporating a pre-metazoan cadhesome “tool kit”

**Fig. 4.**
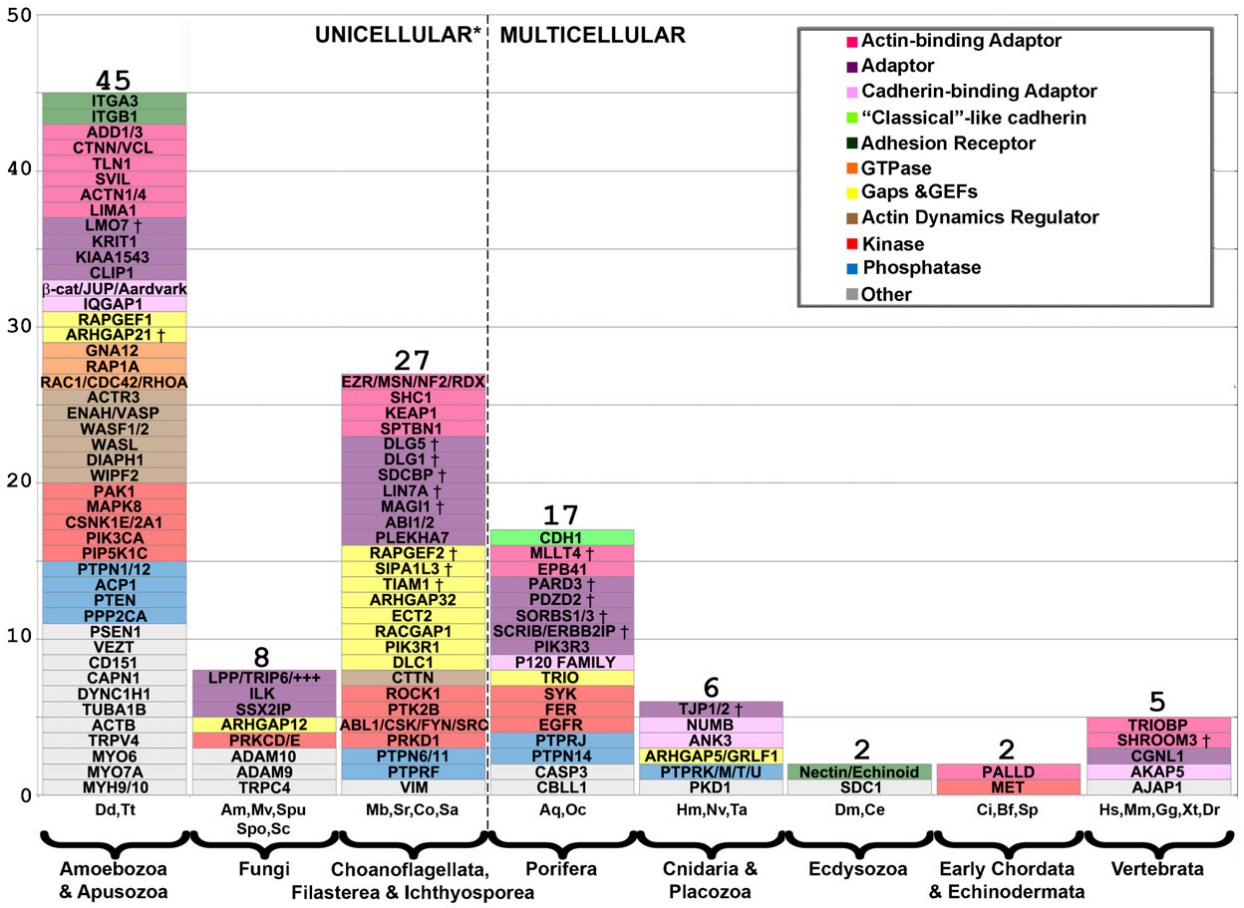
Innovation of cadhesome gene families throughout evolution. The 173 cadhesome components were clustered into a “simplified” cadhesome of 112 families. Novel proteins/protein families (*y*-axis) are plotted according to when – in which phylum/kingdom – a representative of that family first appears, based on the organisms we analyzed (*x*-axis). Some protein families are represented by one member of the family (LPP/++++), with each “+” signifying the existence of an additional family member. Phyla/kingdoms are ordered from left to right by increasing evolutionary age, with the number of novel proteins appearing in each noted above the bars. The dashed line represents the transition from uni- to multicellularity. Gene families are colored according to the legend. PDZ proteins are denoted with a “†”.

Hundreds of proteins interact with the cadherin–catenin “core” of human AJs (Zaidel-Bar, 2013). In order to trace the evolution of this protein network (the cadhesome), we first clustered its components based on similar domain arrangement (e.g. proteins with three LIM domains, such as LPP and TRIP6), which yielded 112 protein families ranging in size from 1 to 18 members (supplementary material Table S4). We plotted each protein family of this “simplified” cadhesome as a function of its implied evolutionary age, i.e. in what phylum a representative analogous protein first appeared (Fig. 4). For example, the origin of “classical”-like cadherin is placed after the transition to multicellularity, while β-catenin/Aardvark is placed before the transition because *Dd*Aardvark was shown to function in a similar manner to β-catenin (Dickinson et al., 2011; Dickinson et al., 2012b). What is strikingly apparent from Fig. 4 is that the majority of cadhesome protein families predate the transition to multicellularity. Summing up the groups to the right of the transition, we found that ∼71% (80/112) of the “simplified” cadhesome predates metazoans.

The pre-metazoan proteins incorporated into the metazoan cadhesome belong to a variety of categories, including kinases, phosphatases, small GTPases and their regulators, actin regulators, and adaptor proteins (Fig. 4). There are 45 families with origins in *D. discoideum*/*T. trahens* or earlier organisms. Though the majority of these ancient proteins are regulatory, there are a few interesting exceptions, e.g. the transmembrane protein VEZT, which is characterized by the Vezatin domain. In vertebrates, VEZT interacts with MYO7A and possibly α-catenin, thereby recruiting MYO7A to AJs where it functions to strengthen cell–cell adhesions (Küssel-Andermann et al., 2000). We found proteins analogous to VEZT, MYO7A, and α-catenin/vinculin in both *Dictyostelium* and *T. trahens*. Though *Dd*VEZT lacks a SMART-defined Vezatin domain, we found it to have two adjacent transmembrane domains followed by a region with high sequence similarity to the other VEZT analogs (supplementary material Fig. S1), which suggests this protein may function in the same network as far back as amoebozoa/apusozoa. VEZT exists in only two other pre-metazoan organisms – *S. rosetta*, and *Spizellomyces punctatus* – indicating a loss in most non-metazoans analyzed.

Noteworthy, the majority of the 27 protein families first appearing in ichthyosporea, filasterea, and choanoflagellata are PDZ-containing adaptor and regulatory proteins (denoted by “†” in Fig. 4), including TIAM1, SIPA1L3, RAPGEF2, Syntenin-1 (SDCBP), LIN7A, MAGI1, Discs large (DLG1), and possibly Discs large 5 (DLG5). According to published transcriptomic data we analyzed, three of these – SDCBP (F2UJY6), DLG1 (F2U0X6), and DLG5 (F2UG17) – are upregulated in *S. rosetta* thecate cells, along with another 23 other PDZ-containing proteins and the *Dictyostelium* polarity network (Fairclough et al., 2013) (supplementary material Tables S1, S2). Of particular interest (see below) is the PDZ-containing protein MAGI. In addition to the MAGI orthologs previously identified in *M. brevicollis* and *C. owczarzaki* (de Mendoza et al., 2010; Ruiz-Trillo et al., 2008), we also found a MAGI analogous protein in *S. rosetta* (F2U2A7). Like *Drosophila* and *Caenorhabditis elegans* MAGI, SMART predicts two WW domains for *Sr*MAGI, which seem to overlap with a predicted GuKc domain (supplementary material Fig. S2). This is in contrast to vertebrate MAGI orthologs in which there is a clear separation between the WW and GuKc domains.

### PDZ–peptide interactions play an important role at the unicellular to multicellular transition

The most basal organism with morphological AJs and a clearly defined interaction between a “classical”-like cadherin and β-catenin is the sponge, *O. carmela*. Fig. 4 demonstrates that the difference in cadhesome content between sponges and pre-metazoans is only 17 protein families, including “classical”-like cadherins with clear catenin-binding tails. Most of the novel metazoan proteins are adaptors, including afadin (MLLT4), ponsin/vinexin (SORBS1/3), PDZD2, Protein 4.1 (EPB41), Par3 (PARD3), and Scribble (SCRIB), many of which bind catenins via their PDZ domains. Importantly, the PDZ-binding motifs of β-catenin and P120 family members are well conserved from sponge to humans, whereas they are absent from all the Aardvark-like proteins we identified in unicellular holozoa, except for *Sa*SARC_01382 (supplementary material Table S3). Except for the cadherins from *Hydra Vulgaris* and *Trichoplax adhaerens*, PDZ-binding motifs are absent from metazoa “classical”-like cadherins outside of bilateria. Despite this, between “classical”-like cadherins and catenins, at least two components of the “core” have PDZ-binding motifs in every metazoan we analyzed. Thus, it appears that novel PDZ–peptide interactions were important innovations at the dawn of metazoa.

**Fig. 5.**
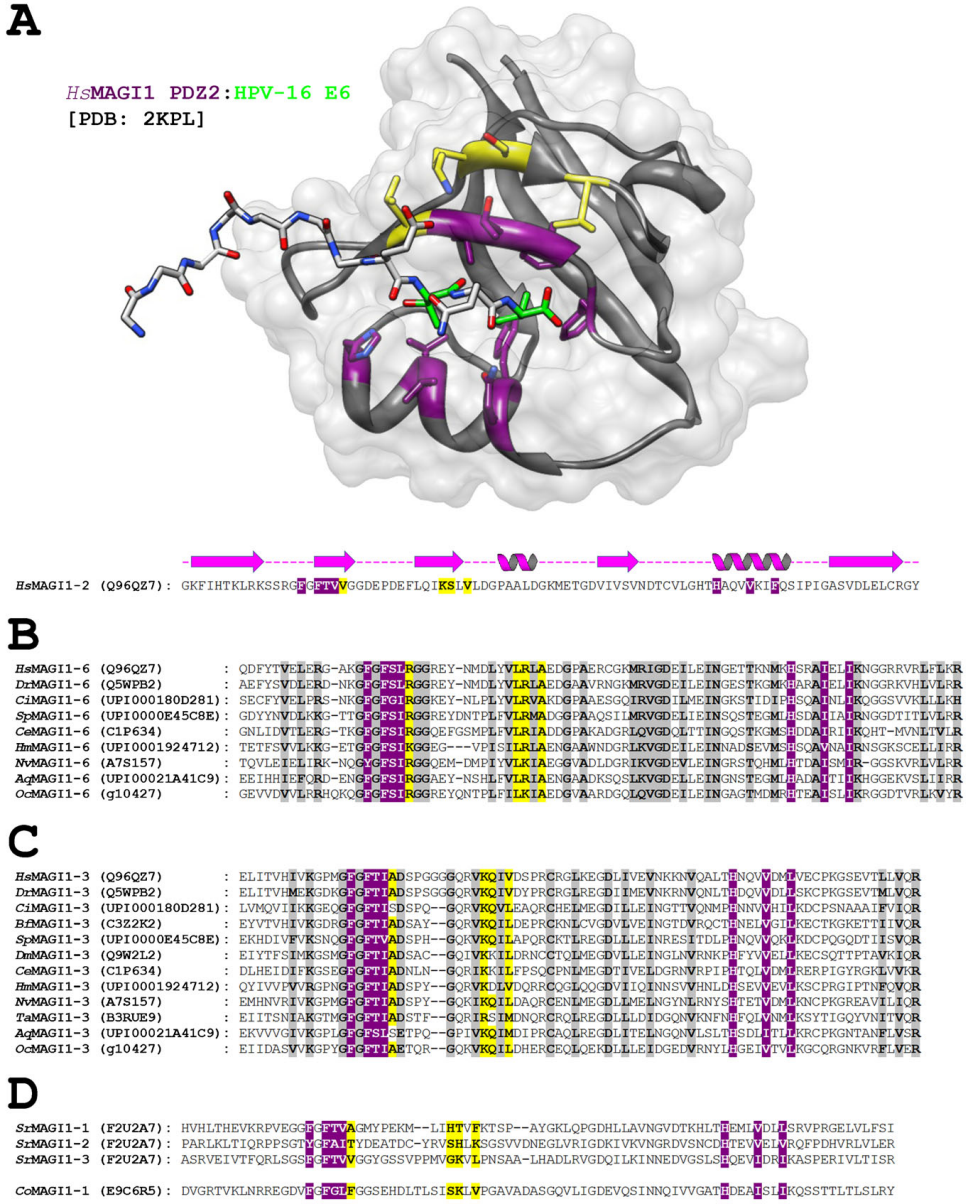
Conservation of selected PDZ domains from MAGI. (A) A peptide derived from the C-terminus of the human papilloma virus E6 protein is shown in complex with human MAGI1 PDZ2 (PDB: 2KPL). E6 residues “0” and “−2” (green) bind in a cleft formed by the PDZ fold: residues well conserved in all PDZ domains (purple), and residues found to be important for defining peptide-binding specificity (yellow) are shown. Also shown are the sequence and corresponding secondary structure of the PDZ domain. (B,C) Sequence alignments for selected PDZ domains from Metazoan MAGI are depicted, with the same colors as in panel A. Analogous proteins are represented by their Uniprot/Compagen identifiers. (D) The PDZ sequences of the pre-metazoan MAGI analogs are depicted, with the same colors as in panels A–C.

The innovation of PDZ-binding motifs in the catenins not only served to link them to novel metazoa PDZ proteins, but also to pre-metazoa PDZ proteins. As an example, vertebrate MAGI1 employs its six PDZ domains to form complexes with E-cadherin, β-catenin, afadin, and PTEN during the formation of cell–cell junctions in epithelial cells (Vogelmann et al., 2005; Kotelevets et al., 2005; Dobrosotskaya and James, 2000). Fig. 5A shows the structure of human MAGI1 PDZ2, bound to a viral peptide, with the residues that define peptide-binding specificity mapped onto the structure (in yellow). As depicted in Fig. 5B, PDZ6, which binds β-catenin (Dobrosotskaya and James, 2000), is well conserved in all metazoa analyzed, including the residues that define specificity (Tonikian et al., 2008), suggesting that the MAGI1-β-catenin link is conserved as far back as sponges. MAGI PDZ3, which recruits PTEN is also well conserved, as is the PDZ-binding motif of PTEN from *O. carmela* (Fig. 5C). Thus, via its multiple PDZ domains, even the most basal metazoan MAGI analogous protein seems capable of integrating multiple proteins, e.g. β-catenin and PTEN, at AJs. This is in stark contrast to the pre-metazoan MAGI proteins – from *C. owczarzaki* and *S. rosetta* – which possesses one and three PDZ domains, respectively, none of which have similarity to *Hs*MAGI PDZ3 or PDZ6 (Fig. 5D). Thus, the innovation of metazoan catenins with PDZ binding motifs is coupled to the appearance in the most basal metazoa of PDZ domains, both in the form of novel proteins, and in new or altered PDZ domains within existing proteins.

### Expansion of the cadhesome in metazoa

**Fig. 6.**
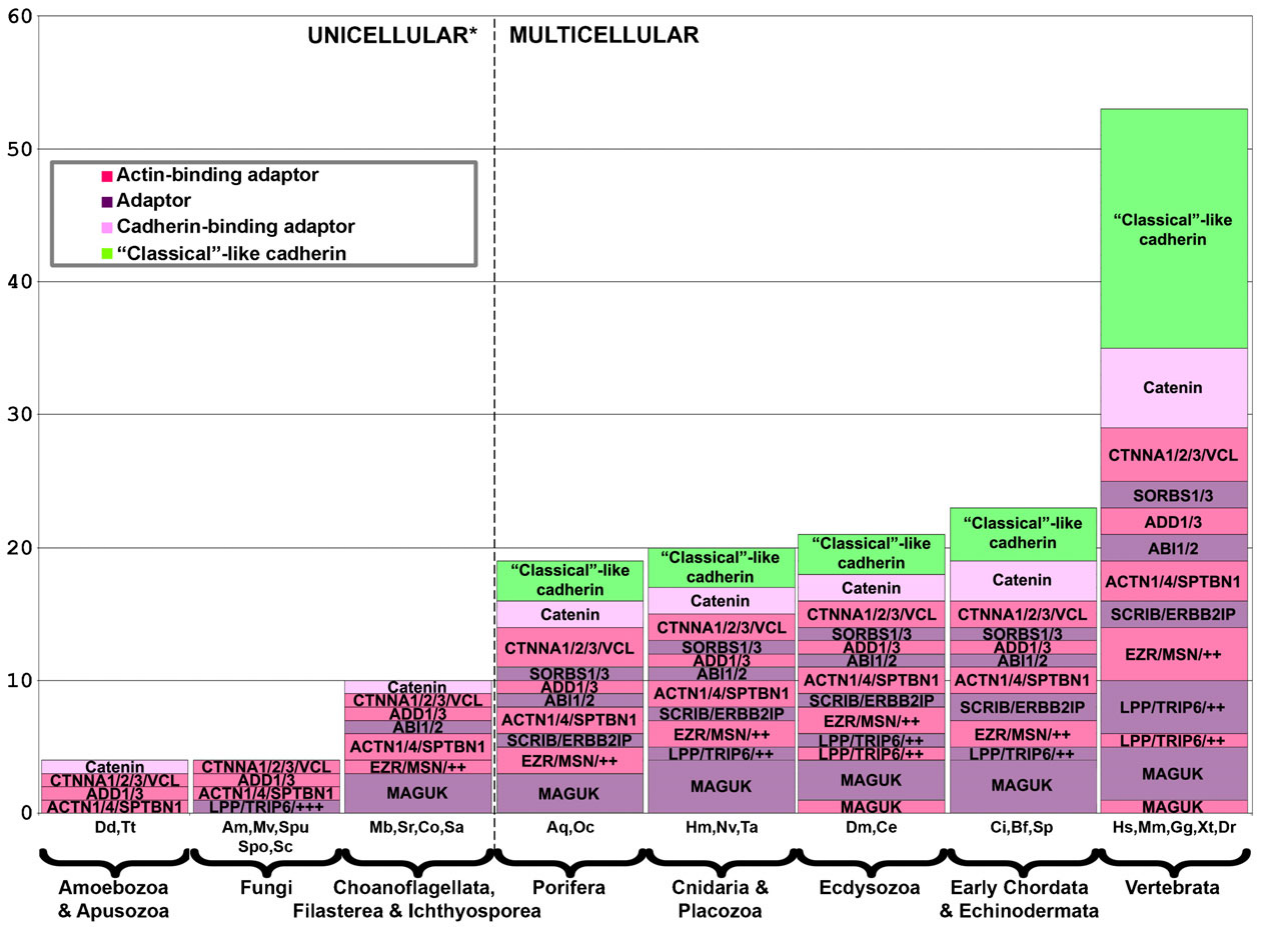
Expansion of cadhesome gene families throughout evolution. “Classical” cadherin-like proteins, cadherin-binding adaptors, actin-binding adaptors, and adaptors are portrayed. These categories were divided up into the same groups as the “simplified” cadhesome, except for ACTN1/4/SPTBN1 and the MAGUK proteins, which were more broadly clustered (see Materials and Methods). The total number of members in each group (*y*-axis) was plotted over evolutionary time (*x*-axis), as represented by the evolutionary ages of the phyla/kingdoms, which are ordered from left to right by increasing age. The dashed line represents the transition from uni- to multicellularity. Gene families are colored according to the legend.

We found that ∼87% (97/112) of the “simplified” cadhesome existed in the most basal metazoa. Fig. 4 shows that only 15 novel proteins or protein families appeared since sponges diverged from the other metazoa, including Tara (TRIOBP), SHROOM3, palladin (PALLD), and paracingulin (CGNL1), all of which are either actin- or myosin-binding proteins. This suggests an increased variety of cytoskeletal attachment. Though only a few novel protein families appeared in higher metazoa, the total number of known cadhesome components nearly doubled, from ∼100 in basal metazoa to ∼170 in vertebrates (supplementary material Fig. S3). To analyze this expansion in more detail, we focused our attention on cadherins, cadherin-binding adaptors, actin-binding adaptors, and other adaptors. Fig. 6 shows the total number of members per group in each phylum, from amoebozoa/apusuzoa to vertebrates. Significant vertebrate expansion is exhibited by all three adaptor groups, including proteins characterized by three LIM domains (LPP, TRIP6, FBLIM1, JUB, and ZYX), which have been implicated in mechano-sensing (Schiller et al., 2011). The most striking example of expansion is experienced by the cadherins. Outside of vertebrates, all metazoa examined have at most three “classical”-like cadherins; within vertebrates there are between 12 and 18 “classical” cadherins of type I and II.

## DISCUSSION

It is now well established that cadherins predate metazoa, existing in choanoflagellata and filasterea (Abedin and King, 2008; Nichols et al., 2006), early unikonts such as the apusozoa, *T. trahens* (identified herein), and earlier eukaryotes such as the plant pathogen, *Pythium ultimum* (Nichols et al., 2012), but only metazoa have cadherins with a clearly conserved JMD and CBD (Fig. 2) (Miller et al., 2013). Our search of pre-metazoa for “classical” cadherins seems to confirm this: we found no clear JMD or CBD outside of metazoa, and no JMD GBM or CBD GBM. It has recently been suggested that cadherin-based adhesion may derive from a cadherin-based bacterial feeding system, incorporated from a unicellular ancestor, and perhaps still present in modern choanoflagellates (Abedin and King, 2008). In this system, choanoflagellate cadherins may bind to extra-cellular receptors on bacteria, which triggers phagocytosis of the prey-bound cadherin, similar in principle to how Listeria triggers endocytosis via E-cadherin in humans (Mengaud et al., 1996). Consistent with this, the choanoflagellate cadherins, *Mb*CDH1 and 2, have been localized to actin-filled microvilli in the feeding collar of *M. brevicollis* (Abedin and King, 2008). Furthermore, transcriptomic analysis has demonstrated that a subset of cadherins is upregulated in *S. rosetta* thecate cells, perhaps linking them to feeding or substrate adhesion (Fairclough et al., 2013). A different set of cadherins is upregulated in colonies, in which cells have been observed to be linked by intercellular bridges and a shared extracellular matrix (Dayel et al., 2011).

**Fig. 7.**
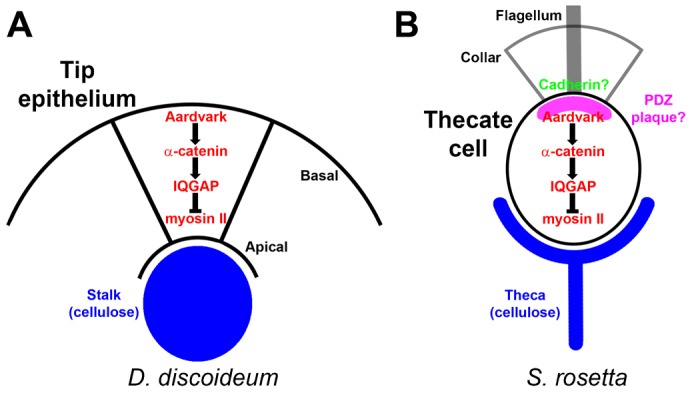
Model of *Dictyostelium* polarity network in *S. rosetta* thecate cells. (A) The proposed organization of the *Dictyostelium* polarity network is depicted for cells of the *Dictyostelium* tip epithelium (Dickinson et al., 2012b). Aardvark is located at the basal end of the cell, with myosin II at the apical end, where cellulose is secreted to form the stalk (in blue). (B) Our hypothetical model of polarity network organization is depicted for *S. rosetta* thecate cells. Analogous to *Dictyostelium* tip cells, the theca (blue), which is comprised of cellulose, is secreted from the “apical” end, where myosin II is located. Aardvark is located at the “basal” end by the feeding collar, where choanoflagellate cadherins are known to localize. Some of these cadherins possess PDZ-binding motifs, so we also placed the theoretical thecate PDZ plaque (magenta) on the basal end with cadherins.

Our search of unicellular holozoa with β-catenin and Aardvark identified at least six Aardvark-like sequences in *S. rosetta*, three in *S. arctica*, and one in *C. owczarzaki* (Fig. 3). When we analyzed recent *S. rosetta* transcriptomics data (Fairclough et al., 2013) we found that one of these Aardvark-like sequences is upregulated in thecate cells, along with protein analogs of α-catenin/vinculin, IQGAP, and myosin II, which together function to establish epithelial-like polarity in *Dictyostelium* (Dickinson et al., 2012a), as well as four membrane-spanning cadherins. As noted above, none of these cadherins have a clear catenin-binding JMD or CBD, but, in theory, this places the “core” elements of AJs together in the same cell type of the same organism. Furthermore, the organization of the *Dictyostelium* polarity proteins (Fig. 7A) (Dickinson et al., 2012b) fits well with a model layout of an *S. rosetta* thecate cell, with Aardvark on the collar end, possibly co-localized with cadherins (Abedin and King, 2008), and myosin II on the opposite end, from which cellulose is secreted to form the theca (Fig. 7B). Though this suggests *S. rosetta* thecate cells are more relevant to metazoa multicellularity than the colonial state, we also note co-upregulation of cadherins and Aardvark-like sequences in colonies, perhaps suggesting a greater diversity in these organisms of the link between cadherins and proteins related to the catenins. It is interesting to note that the Aardvark-like proteins we identified in *S. rosetta* contain Toll/interleukin-1 receptor (TIR) domains, which have a conserved role in environmental sensing and innate immunity (O'Neill, 2000), raising the intriguing possibility that early cell–cell adhesion structures recruited signaling modules from the innate immunity pathway. Of the other unicellular holozoa we analyzed, only *C. owczarzaki* has all the basic elements of the cadherin–catenin “core”, though it has only two cadherins.

**Fig. 8.**
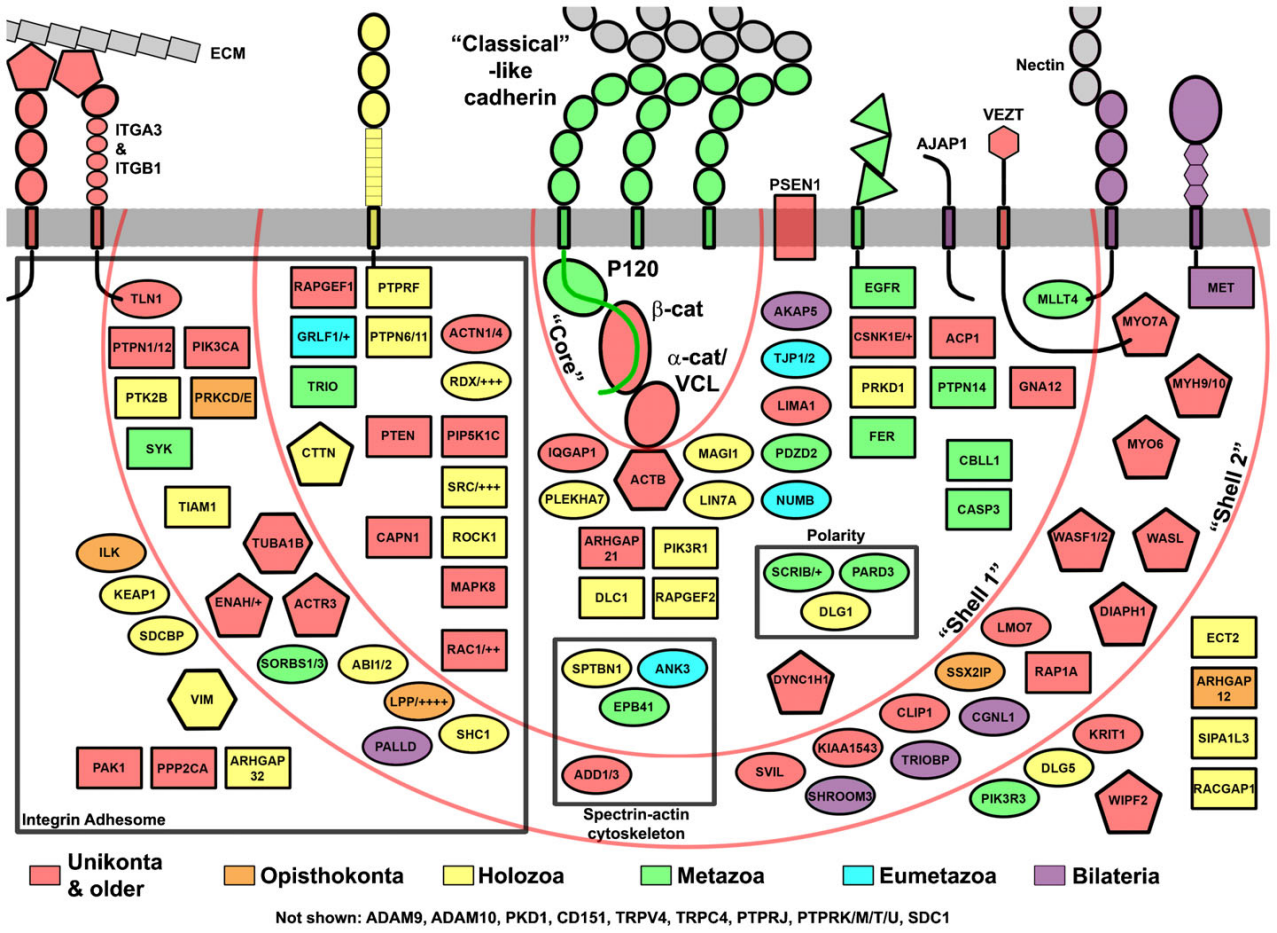
Evolutionary age of cadhesome components. Proteins and protein families of the “simplified” cadhesome are colored according to when in evolution they appeared, and organized based on how they interact with the “classical” cadherin–catenin “core” (central semi-circle). Protein age is distinguished by color: unikont origin or older (red), opisthokont (orange), holozoa (yellow), metazoan (green), eumetazoa (cyan), and bilateria/vertebrata (violet). Some protein families are represented by one member of the family (LPP/++++), with each “+” signifying an additional family member. Components that interact directly with the cytoplasmic tail of cadherin or one of the catenins in vertebrates are located within “Shell 1”; components that interact indirectly with cadherin–catenin – i.e. via components in “Shell 1” – are located within “Shell 2”. All remaining components are located outside the two shells. Component types are distinguished by shape: adaptors (oval), regulatory proteins (rectangle), actin dynamics regulators (pentagon), cytoskeleton (hexagon), with receptors spanning the plasma membrane (and extracellular domains drawn to schematically show their domain structure). Cadhesome receptors are colored based on when in our analysis they appear as full membrane-spanning proteins with both cytoplasmic and extracellular domains. Boxed are components shared with other systems, including the spectrin–actin cytoskeleton, metazoan polarity complexes, and the integrin adhesome.

Many studies have demonstrated that AJs are more than simply the cadherin–catenin “core”. Searching the literature, we previously found over 170 proteins reported to be associated with the “core”. We call this network of proteins and their interactions the cadhesome (Zaidel-Bar, 2013). To determine when in evolution cadhesome proteins first appeared we first clustered similar proteins into families, yielding the “simplified” cadhesome, and then determined in what phyla a representative of each family first appeared (Fig. 4). This is depicted in graphical form in Fig. 8, where evolutionary age is color-coded, function is shape-coded, and position is a function of whether the human protein interacts directly or indirectly with the “core”. As evident in Fig. 8, protein families with pre-metazoa origins (colored red, orange, and yellow) make up ∼70% of this “simplified” cadhesome, and many of these interact with the “core” (innermost shell). That so many ancient proteins interact with the “core” directly (“shell 1”), coupled with the pre-metazoa origins of the catenins, seems to suggest that much of the AJ regulatory network may have been in place prior to metazoa. This helps to explain how the transition from uni- to multicellular life forms could take place, but it also elicits important questions: for example, what was the function of cadhesome proteins before AJs existed? How were so many pre-existing proteins and networks incorporated and integrated by so few novel proteins?

Forty proteins or protein families of the “simplified” cadhesome (black box in Fig. 8) overlap with the similarly compiled integrin adhesome (Zaidel-Bar and Geiger, 2010; Zaidel-Bar et al., 2007; Zaidel-Bar, 2009). According to our analysis, 35 of these 40 families predate metazoa. As focal adhesions predate AJs (Sebé-Pedrós et al., 2010), inclusion of these components in cell–matrix adhesion may explain the pre-metazoa role of some cell–cell adhesion components. Other cadhesome components may have performed similar roles in pre-metazoa, albeit under very different circumstances. Vertebrate VEZT serves to link the cadherin–catenin “core” to MYO7A, thereby strengthening adhesions (Küssel-Andermann et al., 2000), but it is also crucial for Listeria entry into epithelial cells (Sousa et al., 2004). We also found VEZT and MYO7A in *S. rosetta* and *Dictyostelium* where they may serve to internalize bacteria for feeding or other non-infection purposes. Thus, by recruiting VEZT to AJs, the first metazoa may have also recruited MYO7A. Indeed, it may have been possible to incorporate small multi-protein networks into the cadhesome, by way of only one of the proteins in the complexes, e.g. Aardvark or VEZT.

We find that over half of the novel proteins in unicellular holozoa and basal metazoa were modular adaptor proteins, specifically PDZ adaptors (Figs 4 and 5). This innovation occurred concurrently with the development in metazoa of catenins with PDZ-binding motifs (supplementary material Table S1). In theory, these two innovations may have served to link the cadherin–catenin “core” to many other proteins in the cadhesome, thereby integrating multiple proteins at the origin of metazoa. The principle of domain modularity is well appreciated in the context of evolution: domain shuffling, the addition of pre-existing domains to proteins, and the innovation of novel domains, all open up new sets of interactions and functions (Pawson and Nash, 2003; Jin et al., 2009). Indeed, many of these processes seem to have occurred in choanoflagellates (King et al., 2008; Lim and Pawson, 2010; Fairclough et al., 2013). Judging by the overall rise in PDZs around the transition to multicellularity, and their use as protein–protein interaction modules in adaptors from large complexes, e.g. the post-synaptic density, PDZ domains seem well suited to this purpose (Fahey and Degnan, 2010; Sakarya et al., 2010; Sakarya et al., 2007). Furthermore, few changes are required to transform the unstructured C-terminus of a given protein into a PDZ-binding motif (Kim et al., 2012), as seems likely to have taken place with many cadherins (Demontis et al., 2006), including some in choanoflagellates (supplementary material Table S4). Interestingly, we found that many PDZ domains, including three from the cadhesome, are upregulated in *S. rosetta* thecate cells together with specific cadherins and the *Dictyostelium* polarity network. This may hint at the existence of a pre-metazoa PDZ–protein plaque, similar to that of AJs or the post-synaptic density, which may interact with choanoflagellate cadherins, some of which have PDZ-binding motifs (Fig. 7B). What is clear from our analysis is that multiple-PDZ adaptor proteins were a critical development at the dawn of multicellularity, as they allowed for the incorporation and integration of pre-metazoa proteins in the first metazoa.

The primary mode of cadhesome expansion in the 600 million years since the emergence of metazoa was gene duplication (Fig. 6). This expansion likely reflects both a growing need for complexity, within and between cells, and the increased specialization of cells and tissue; furthermore, it coincides with a well-known whole-genome duplication event in early vertebrates (Lundin, 1999). In many cases, the paralogs conserve the primary functionality of the original component, while gaining or losing secondary functions. For example, all members of the vertebrate P120 family bind to the JMD of “classical” cadherins. However, unlike the most ancestral of the P120 family (Zhao et al., 2011; Carnahan et al., 2010), P120 has no PDZ binding motif at its C-terminus, which suggests it has been decoupled from PDZ adaptor proteins and their functionalities. The functional diversity of catenins is thus enabled by modularity: the N- and C-termini of the P120 family are free to mutate and sample new functions, while the cadherin-binding ARM repeats remain relatively unchanged.

The evolution of the cadhesome network appears to be the product of a variety of genetic processes: incorporation, integration, innovation, and expansion. In elucidating these processes, we have identified a number of interactions conserved in sequence and possibly structure across hundreds of millions of years, and constructed a plausible evolutionary history of cadherin-based adhesion systems. This reconstruction makes specific testable predictions by evolutionary inference. For example, we would expect the MAGI and β-catenin analogs from *O. carmela* to bind each other. The next step will be to experimentally test these hypotheses, as has been done for related questions in *D. discoideum* and the sponge, *O. carmela* (Dickinson et al., 2011; Nichols et al., 2012). Much of the integrin adhesome (81) has been recently validated by proteomic approaches (reviewed by Geiger and Zaidel-Bar, 2012). On top of validating a systems-level approach for studying adhesion junctions, hundreds of additional components were identified, suggesting our current view of junctions is still overly simplistic. As more experimental data will expand our view of the cadherin adhesome, an evolutionary perspective, such as is presented here, coupled with advances in methods such as transcriptomics and proteomics, will undoubtedly help make sense of the complex biology presented to us.

## Supplementary Material

Supplementary Material
