# Supplementary material for: Pre-metazoan origins and evolution of the cadherin adhesome
Source: Biol Open. 2014 Nov 13;3(12):1183–95. doi: 10.1242/bio.20149761 (PMC4265756; doi:10.1242/bio.20149761)
Supplement: Supplementary Material [file supp_3_12_1183__index.html]

Pre-metazoan origins and evolution of the cadherin adhesome — Supplementary Material 

# Pre-metazoan origins and evolution of the cadherin adhesome

## bio.20149761 Supplementary Material

**Files in this Data Supplement:**

- Supplementary Material - Paul S. Murray and Ronen Zaidel-Bar doi: 10.1242/bio.20149761
- Table S4
- Table S5
- Table S6
- Table S7
