# Supplementary material for: Pre-metazoan origins and evolution of the cadherin adhesome
Source: Biol Open. 2014 Nov 13;3(12):1183–95. doi: 10.1242/bio.20149761 (PMC4265756; doi:10.1242/bio.20149761)
Supplement: Supplementary Material [file supp_bio.20149761_bio.20149761-s1.pdf]

Biology Open (2014) 000, 1–13 doi:10.1242/bio.20149761

Supplementary Material

Paul S. Murray and Ronen Zaidel-Bar doi: 10.1242/bio.20149761

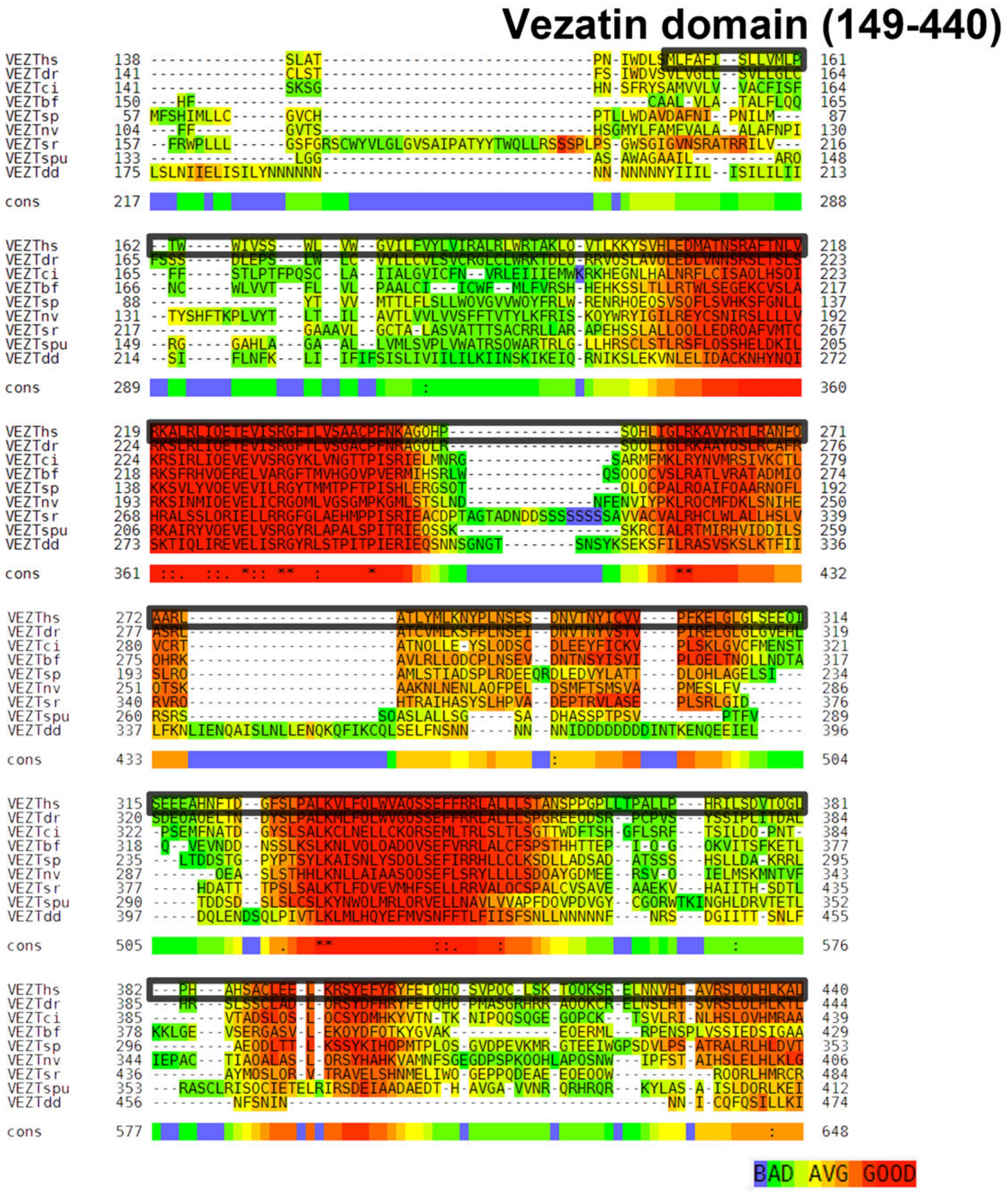

Fig. S1. Alignment of VEZT across unikonts. The PSI-Coffee alignment of VEZT is portrayed for all the VEZT orthologs we analyzed (except *T. trahens* VEZT), from humans at the top (*Hs*VEZT), down to *Dictyostelium* VEZT (*Dd*VEZT). The alignment is colored based on sequence conservation, from blue (poorly aligned) to red (well aligned). Boxed in black is the SMART-predicted Vezatin domain (residues 149–440) for *Hs*VEZT.

Biology Open

Biology Open (2014) 000, 1–13 doi:10.1242/bio.20149761

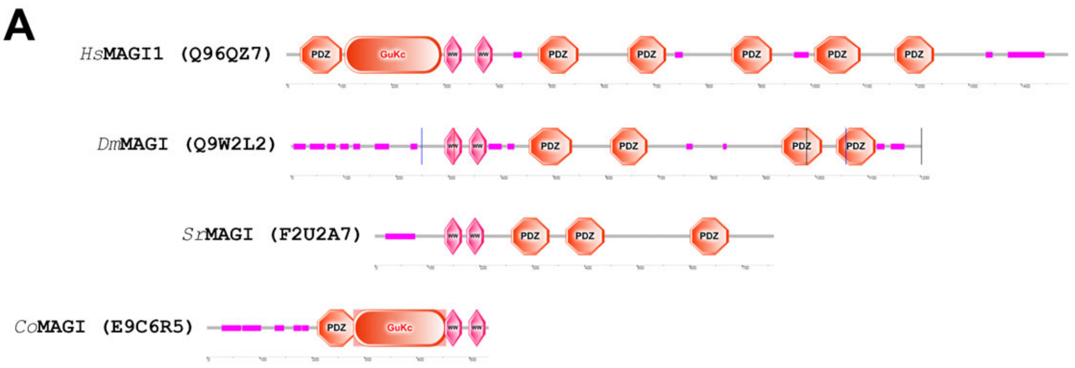

B

	PDZ1	GuKc	WW	WW	PDZ2	PDZ3	PDZ4	PDZ5	PDZ6
*Hs*MAGI1	0.015	1.8E-46	9.6E-11	4.4E-09	3.7E-18	2.5E-15	4.6E-19	2.3E-23	1.6E-20
*Dm*MAGI	N/A	3.0E-28 (BLAST)<br>N/A	N/A<br>6.6E-09	N/A<br>6.8E-06	3.7E-17	4.9E-09	5.8E-16	1.70E-18	N/A
*Sr*MAGI	N/A	3.0E-14 (BLAST)<br>N/A	N/A<br>1.3E-03	N/A<br>1.0E-04	2.2E-12	2.9E-12	1.1E-19	N/A	N/A
*Co*MAGI	1.9E-11	1.4E-03	1.3E-07	2.8E-06	N/A	N/A	N/A	N/A	N/A

Fig. S2. Novel MAGI protein in the choanoflagellate, *S. rosetta*. (A) SMART-predicted domains are depicted for the MAGI orthologs from humans (*Hs*MAGI1), *Drosophila* (*Dm*MAGI), *S. rosetta* (*Sr*MAGI), and *C. owczarzaki* (*Co*MAGI). Included are UniProt identifiers. (B) SMART domain E-values are depicted for the domains of the four MAGI orthologs in part A. Included are the predicted GuKc domains of DmMAGI and SrMAGI, which overlap with the WW domains in these sequences.

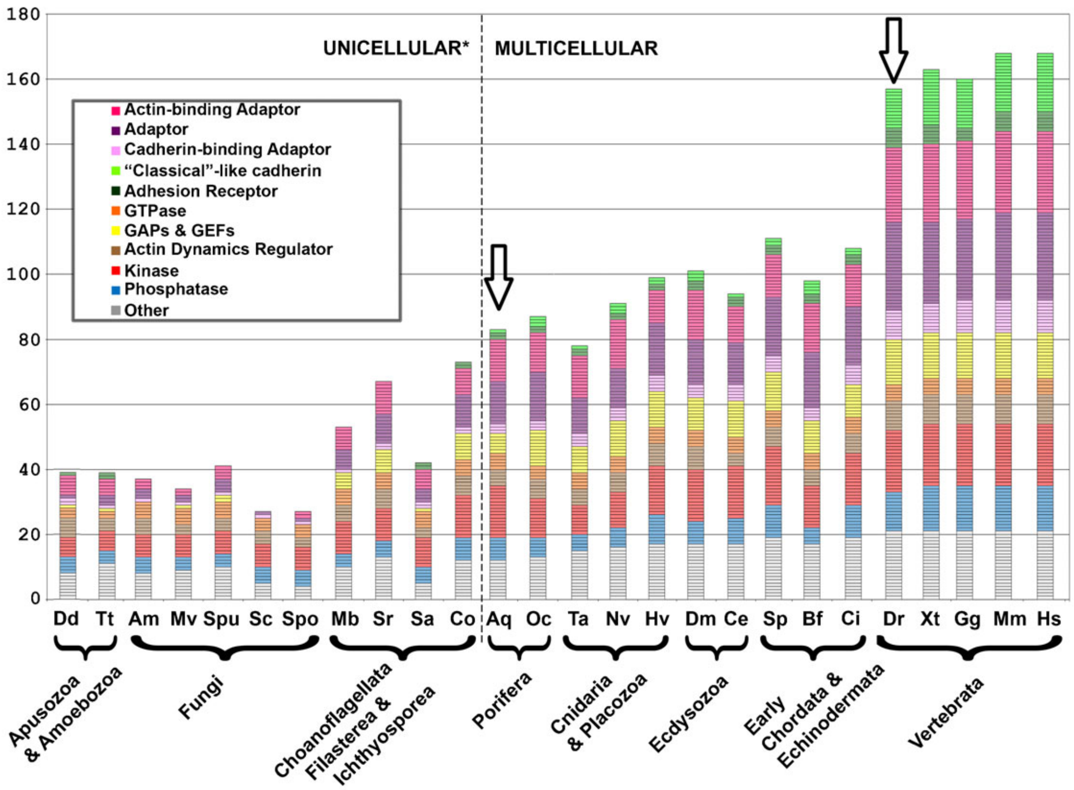

Fig. S3. Cadhesome orthologs throughout evolution. Plotted are the total number of cadhesome orthologs (*y*-axis) in each of the twenty-five species we searched, plus humans (*x*-axis), with the phyla/kingdoms ordered from left to right by increasing evolutionary age. Each ortholog hit is colored according to its general function, as denoted in the legend. The dashed line between protozoa and porifera represents the transition to multicellularity. Unicellular is starred (*) because some of the organisms to the right of the dashed line, e.g. *D. discoideum*, have aggregate, multicellular-like states. The two arrows denote the two major jumps in total cadhesome content.

Biology Open (2014) 000, 1–13 doi:10.1242/bio.20149761

Table S1. *S. rosetta* cadherin, Aardvark-like, and cadhesome genes upregulated in thecate, swim, and colony cells

	THECATE	SWIM	COLONY
Membrane-spanning cadherins	F2TZ15 (PTSG_01815)<br>F2UNP4 (PTSG_12970)<br>F2UFT1 (PTSG_12444)<br>F2TZE6 (PTSG_11807)	F2TZ07 (PTSG_01807)<br>F2U4J1 (PTSG_03209)<br>F2TZJ3 (PTSG_01986)<br>F2UJP7 (PTSG_08442)	F2UDL2 (PTSG_06068)<br>F2UFV3 (PTSG_06458)
Aardvark-like	F2UQ79 (PTSG_10731)		F2UNI1 (PTSG_09915)<br>F2UQC3 (PTSG_10776)
Cadhesome	**CTNNA1/VCL** (F2UM91, PTSG_09306)<br>**IQGAP1** (F2TWA6, PTSG_00372)<br>**MYH10** (F2U9L1, PTSG_04749)<br>**DLG1** (F2U0X6, PTSG_01141)<br>**DLG5** (F2UG17, PTSG_12450)<br>**SDCBP** (F2UJY6, PTSG_08532)<br>**SVIL** (F2U4N4, PTSG_03254)<br>**RAPGEF2** (F2UMV8, PTSG_09152)	**RAPGEF1** (F2UC42, PTSG_06158)	**MAGI1** (F2U2A7, PTSG_02471)<br>**RACGAP1** (F2TYP6, PTSG_01699)<br>**TRPC4** (F2UPF2, PTSG_10078)<br>**ECT2** (F2U0E1, PTSG_11734)

Biology Open (2014) 000, 1–13 doi:10.1242/bio.20149761

Table S2. ***S. rosetta*** **genes upregulated in thecate, swim, and colony cells**

	THECATE	SWIM	COLONY
Protein (Gene)	F2U0X6 (PTSG_01141)	F2UEX4 (PTSG_06827)	F2U2A7 (PTSG_02471)
Domains	L27, PDZ [3], SH3, GuKc	PDZ	WW, WW, PDZ [3]
Re-BLAST	DLG4 (7E-122)	DLG4 (2E-07)	MAGI3 (6E-25)
Protein (Gene)	F2UJY6 (PTSG_08532)	F2UKW9 (PTSG_12804)	F2UG60 (PTSG_06562)
Domains	PDZ, PDZ	PDZ [6]	PDZ [5]
Re-BLAST	SDCBP (4E-74)	DLG4 (2E-09)	DLG4 (7E-17)
Protein (Gene)	F2UG17 (PTSG_12450)	F2U9V4 (PTSG_04844)	F2UIM1 (PTSG_07410)
Domains	PDZ [4], SH3, GuKc, PDZ, GuKc, PDZ [12]	PDZ, coiled-coil	C2, RhoGEF, PDZ [4]
Re-BLAST	DLG2 (9E-69)	MPDZ (4E-04)	PDZK1 (4E-14)
Protein (Gene)	F2U242 (PTSG_02407)	F2UE09 (PTSG_07089)	F2U561 (PTSG_03427)
Domains	PDZ	PDZ [3]	Coiled-coil, PDZ, PDZ
Re-BLAST	SNTA1 (4E-18)	DFNB31 (3E-34)	MPDZ (2E-08)
Protein (Gene)	F2UG16 (PTSG_12449)	F2U7H8 (PTSG_04002)	F2UNZ1 (PTSG_09760)
Domains	B41, FERM_C, PDZ	PDZ, RA	Tryp_SPc, PDZ
Re-BLAST	EPB41L2 (2E-31)	FRMPD4 (9E-19)	HTRA1 (7E-13)
Protein (Gene)	F2ULE3 (PTSG_09577)	F2UFT3 (PTSG_06438)	F2U2T2 (PTSG_02611)
Domains	PDZ	Coiled-coil, PDZ, PTPc, coiled-coil, PDZ, coiled-coil [3]	PDZ, coiled-coil
Re-BLAST	–	CEP164 (0.001)	SLC9A3R1 (5E-04)
Protein (Gene)	F2UDS0 (PTSG_07006)	F2URD4 (PTSG_10914)	F2USL6 (PTSG_11162)
Domains	PTB, PDZ	PDZ, STYKc, RGS, RBD	Coiled-coil, PDZ
Re-BLAST	DLG5 (4E-05)	RGS21 (5E-17)	AZI1 (0.001)
Protein (Gene)	F2URN7 (PTSG_10548)		
Domains	PDZ, GuKc, PDZ, PTPc		
Re-BLAST	PTPRJ (1E-58)		
Protein (Gene)	F2TY17 (PTSG_00978)		
Domains	FCH, coiled-coil, PDZ		
Re-BLAST	ABI2 (0.001)		
Protein (Gene)	F2U904 (PTSG_04921)		
Domains	PDZ, coiled-coil, PDZ, coiled-coil, coiled-coil		
Re-BLAST	SLC9A3R1 (8E-20)		
Protein (Gene)	F2UA87 (PTSG_05375)		
Domains	ANK, ANK, ANK, PTPc, coiled-coil		
Re-BLAST	PTPRS (6E-40)		
Protein (Gene)	F2UNC8 (PTSG_09863)		
Domains	L27, L27, PDZ, SH3, GuKc		
Re-BLAST	MPP5 (2E-62)		
Protein (Gene)	F2U5J3 (PTSG_03839)		
Domains	TM, TNFR [3], Cohesin, TNFR [8], TM, PDZ		
Re-BLAST	TMEM132B (3E-11)		
Protein (Gene)	F2U6B0 (PTSG_03689)		
Domains	SH2, SH2, PDZ		
Re-BLAST	SH2B1 (4E-08)		
Protein (Gene)	F2UHH6 (PTSG_07692)		
Domains	SAM, PDZ, Drmip_Hesp, PDZ, PDZ, PH		
Re-BLAST	CNKSR2 (2E-20)		
Protein (Gene)	F2UAJ7 (PTSG_12260)		
Domains	SH3, PH, PDZ		
Re-BLAST	PREX2 (1E-15)		
Protein (Gene)	F2TX61 (PTSG_00679)		
Domains	PDZ [7], coiled-coil, PDZ [4]		
Re-BLAST	NUDT9 (7E-24)		
Protein (Gene)	F2UPA7 (PTSG_10028)		
Domains	PDZ [15], coiled-coil [3], GAS2		
Re-BLAST	GRIP2 (5E-15)		
Protein (Gene)	F2TYR9 (PTSG_01722)		
Domains	PDZ, SH2, coiled-coil		
Re-BLAST	INCENP (0.86)		
Protein (Gene)	F2UM47 (PTSG_09073)		
Domains	Coiled-coil [4], PDZ		
Re-BLAST	–		
Protein (Gene)	F2UCV9 (PTSG_05818)		
Domains	PDZ, LIM, LIM		
Re-BLAST	PDLIM7 (3E-14)		
Protein (Gene)	F2UT03 (PTSG_13168)		
Domains	PDZ [3], coiled-coil, WW		
Re-BLAST	STXBP4 (1E-11)		

Biology Open (2014) 000, 1–13 doi:10.1242/bio.20149761

Table S2. Continued.

	THECATE	SWIM	COLONY
Protein (Gene)	F2U641 (PTSG_03619)		
Domains	PDZ, Cation_ATPase_N, E1-E2_ATPase, Hydrolase_3, TM, Cation_ATPase_C		
Re-BLAST	ATP2B4 (0)		
Protein (Gene)	F2U4T2 (PTSG_03306)		
Domains	Coiled-coil [2], PDZ [6]		
Re-BLAST	DLG2 (3E-49)		
Protein (Gene)	F2TWZ0 (PTSG_00608)		
Domains	Coiled-coil, coiled-coil		
Re-BLAST	–		
Protein (Gene)	F2UFT0 (PTSG_06434)		
Domains	PDZ, PDZ, SMR		
Re-BLAST	INADL (8E-05)		

Biology Open (2014) 000, 1–13 doi:10.1242/bio.20149761

Table S3. PDZ-binding motifs in metazoan catenins, PTEN, and related protozoan proteins

Species	β-catenin and Aardvark	P120 family	α-catenin and VCL	"classical"-like cadherin	PTEN
Hs	AWFDTDL (P35222) [CTNNB1]	ASPDSWV (Q9UQB3) [CTNND2]	FKAMDSI (P35221) [CTNNA1]	PREELLY (P33151) [CDH5]	HTQITKV (P60484) [PTEN]
Dr	AWFDTDL (Q7ZU14)	ASPDSWV (B0V2S9)	FKAMDSI (Q9PVF8)	SDSDSSY (Q68SP4)	HAQITKV (Q6TGR7)
Ci	PWLDTDL (Q9NL44)	EGLDSWV (UPI0002B8DA34)	*None*	GRRKTAL (F6WV54)	*None*
Bf	AWFDTDL (C3ZT37)	*Fragment*	FSSRDTF (C3Y5U8)	SEQELRI (C3XX25)	*None*
Sp	AFFDTDL (W4Y0D3)	PAGDSWV (UPI000265335C)	*None*	NDDEIQI (UPI0000E46DFB)	VTEITHV (UPI0000E466DC)
Dm	AWYDTDC (P18824)	*None*	QSPADAV (P35220)	DDQGWRI (Q24298)	SGESTYL (Q9Y0B5)
Ce	NWYDTDL (O44326)	NIDDSWV (Q9U308)	KPMWSNF (Q8MPS2)	*None*	FDQAIYL (G5EE01)
Hv	GWFDPDL (T2MGP6)	*Fragment*	*N/A*	DDSEEDV (UPI0002B47A87)	*None*
Nv	PLYDTDL (A7SKL9)	DASDSWV (A7S9K2*)	RRKPDFY (A7RIF5*)	*None*	*None*
Ta	EVKFILL **(B3RR98)**	*None**	*None**	PSKSENV (B3RVM9*)	TLFTDCL (B3S4N1)
Aq	GWIDTDL (E2IJA6)	GAIDSWV (UPI00021A402E)	MGENDFL (†)	*None*	EVESVAV (UPI00021A3DFE)
Oc	HFFDTDL (g9376.t1)	PVEDSWV (g1255.t1)	PEEDEWV (g3385.t1)	*None*	WGQTSKV (g1443.t1)
Sr	*None*	*N/A*	*None*	*N/A*	GLPISDV (F2URQ0)
Mb	*N/A*	*N/A*	*None*	*N/A*	GLPISDV (A9V8Q5)
Co	*None*	*N/A*	*None*	*N/A*	*None*
Sa	LCTCTYV (SARC_01382)	*N/A*	RNRDCKF (SARC_06473)	*N/A*	*Fragment*
Dd	*None*	*N/A*	*None*	*N/A*	*None*

If an ortholog for a given protein exists in an organism, and it has a PDZ-binding motif, the C-terminal seven residues are listed, with the PDZ class-defining residues in green; also listed are the Uniprot, Compagen, and Origins of Multicellularity database identifiers. Identifiers denoted by a "*" and "†" are fragments, but the full-length sequence was obtained from Hulpiau and van Roy (Hulpiau and van Roy, 2011) and Fahey and Degnan (Fahey and Degnan, 2010), respectively. "None" indicates an ortholog exists, but has no PDZ-binding motif. "Fragment" indicates an ortholog exists, but its C-terminus is truncated. "N/A" indicates no ortholog exists in this organism.

Tables S4-S7. See supplementary webpage.

Biology Open
